# The influence of the dispersion method on the electrical properties of vapor-grown carbon nanofiber/epoxy composites

**DOI:** 10.1186/1556-276X-6-370

**Published:** 2011-05-04

**Authors:** Paulo Cardoso, Jaime Silva, Donald Klosterman, José A Covas, Ferrie WJ van Hattum, Ricardo Simoes, Senentxu Lanceros-Mendez

**Affiliations:** 1 Center/Department of Physics, University of Minho, Campus de Gualtar, 4710-057 Braga, Portugal; 2 IPC/I3N--Institute for Polymers and Composites, University of Minho, Campus de Azurém, 4800-058 Guimarães, Portugal; 3 Chemical & Materials Engineering, University of Dayton, 300 College Park, Dayton, OH 45469-0246, USA; 4 School of Technology, Polytechnic Institute of Cávado and Ave, Campus do IPCA, 4750-810 Barcelos, Portugal

## Abstract

The influence of the dispersion of vapor-grown carbon nanofibers (VGCNF) on the electrical properties of VGCNF/Epoxy composites has been studied. A homogenous dispersion of the VGCNF does not imply better electrical properties. In fact, it is demonstrated that the most simple of the tested dispersion methods results in higher conductivity, since the presence of well-distributed nanofiber clusters appears to be a key factor for increasing composite conductivity.

PACS: 72.80.Tm; 73.63.Fg; 81.05.Qk

## Introduction

Epoxy resins have a wide range of applications in materials science [1]. By incorporating high aspect ratio fillers like carbon nanotubes (CNT) [2] or vapor-grown carbon nanofibers (VGCNF) [3], the epoxy mechanical and electrical properties are enhanced and the range of applications is extended. The VGCNF electrical and mechanical properties are relatively lower than those obtained with CNT but, on the other hand, they have significant lower cost and are more easily available [3]. VGCNF can be prepared with diameters in the nanometer scale, resulting in high aspect ratios such as the Pyrograf^® ^III nanofibers (Applied Sciences Inc, Cedarville, OH, USA), which are a highly graphitic form of VGCNF with stacked-cup morphology [4].

The focus of recent research related to VGCNF/epoxy composites has been on the development of processing methods able to generate a homogenous dispersion of the VGCNF within the polymer matrix. For instance, Allaoui et al. [5] prepared VGCNF/epoxy composites using a combination of ultrasonication and mechanical mixing, concluding that the composite conductivity can be attributed to the formation of a tunneling network with a low percolation threshold (0.064 wt%). One of the early works with VGCNF/epoxy revealed that the degree of VGCNF dispersion is relevant for the composite mechanical strength [6]. The authors dispersed the VGCNF via acetone solvent/epoxy solution and mixing. The mechanical properties of VGCNF/epoxy composites were also studied by Zhou et al. [7]. The loading effect on the thermal and mechanical properties of the composites was investigated by dispersing the VGCNF through high-intensity ultrasonication. In turn, Prasse et al. [8] used sonication and conventional stirring to disperse the VGCNF. Anisotropy has an effect on the electrical properties: composites with VGCNF preferentially parallel to the electric field show lower electrical resistance and higher dielectric constant. This effect can be explained by the formation of a capacitor network, as demonstrated by Simões et al. [9,10] for CNT/polymer composites. Furthermore, studies of systems such as VGCNF/poly(vinylidene fluoride) demonstrated that the matrix properties, such as the crystallinity or phase type, also influence the type of conduction mechanism in VGCNF/polymer composites [11]. In a previous study [12], the electrical properties of VGCNF/epoxy composites prepared by simple hand mixing were studied, and it was confirmed that conductivity is due to the formation of a tunneling network. As stated before, the VGCNF homogenous dispersion in the matrix is important for mechanical properties, but as discussed in [12], a good cluster distribution seems to be more significant for electrical properties. In fact, as discussed in [3], a good filler distribution is not suitable for electrostatic discharge applications due to static charge build up. Also related to our study, Aguilar et al. [13] has experimentally demonstrated that multiwall carbon nanotube agglomerations at the micro-scale induce higher values for the electrical conductivity in MWCNT/polymer films.

This study focuses on the influence of the dispersion method on the overall electrical properties of a VGCNF/epoxy composite. Four methods were used for the VGCNF dispersion, and the conductivity and dielectric constant of each composite were measured. The resulting dispersion level in each case was analyzed using scanning electron microscopy (SEM) images.

## Experimental

The VGCNF Pyrograf III™, PR-19-LHT-XT, were supplied by Applied Sciences, Inc (Cedarville, OH, USA). The epoxy resin was Epikote™ Resin 862 and the curing agent was Ethacure 100 Curative, supplied by Albemarle. Samples with Epon Resin 862 from Hexion Specialty Chemicals and Epikure W from Resolution Performance Products as a curing agent were also used. The two types of resins and curing agents share the same chemical abstract service (CAS). The weight ratio of resin to curing agent was 100:26.4. The dispersion of the VGCNF in the epoxy resin was performed by four different methods: Method 1: hand mixing with a Haeger blender for 2 min [12], where the velocity field and stress levels should generate a predominantly distributive mixing of the clusters; Method 2: one pass extrusion through a Capillary Rheometer fitted with a series of rings with alternating directions [14], where the strong extensional fields are anticipated to result in a good filler dispersion but limited cluster distribution ; Method 3: roll milling (using a Lehmann 3 roll miller) for 5 min, with a gap of 25.4 μm between the first and second rolls and 600 r.p.m. for the third roll, which is expected to result in a good filler dispersion and a relatively good cluster distribution; Method 4: a planetary-type Thinky ARE-250 mixer, at revolution and rotation speeds of 2000 and 800 rpm, respectively, for 10 min, which should ensure a good cluster distribution. In all cases, the resin and curing agent were pre-mixed by hand [11]. After mixing, all samples were subjected to a 20-mbar pressure, then cast into a mold and cured at 80 and 150°C for 90 min each. Composites with VGCNF concentrations of 0.1, 0.5, 1.0, and 1.5 wt% were prepared, corresponding to volume fractions of 0.0006, 0.003, 0.006, and 0.009, respectively. The samples were rectangular bars with 1 × 10 × 70 mm. VGCNF dispersion in the matrix was investigated by observing surface and cross section images by SEM Phillips X230 FEG. The volume d.c. electrical resistivity of the samples was obtained using the two-probe method, by measuring the characteristic *I*-*V *curves at room temperature with a Keithley 6487 picoammeter/voltage source. The samples were coated on both sides by thermal evaporation with circular Al electrodes of 5-mm diameter. The current and voltage were measured and the resistivity was calculated taking into account geometric factors. The capacity and tan δ, dielectric loss, were measured at room temperature in the range of 500 Hz to 1 MHz with an applied signal of 0.5 V with an automatic Quadtech 1929 Precision LCR meter. The real component of the dielectric function *ε*ε was obtained from the measurement of the capacity and geometrical factors.

## Results and discussion

**Figure 1:**
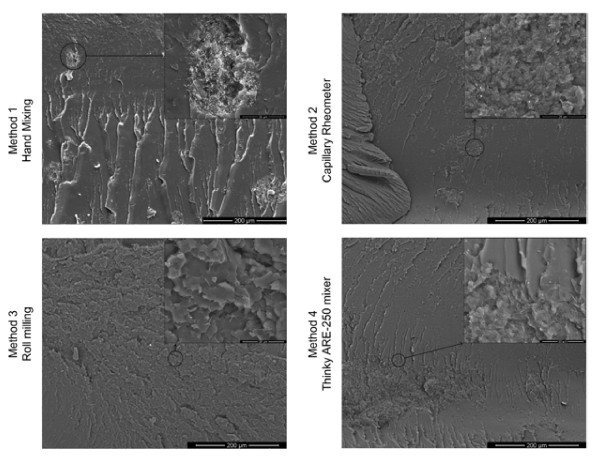
**Cross section SEM images for the 0.006 volume fraction samples**.

The level of VGCNF distribution and dispersion in the matrix achieved by the four preparation methods was estimated from SEM images; see Figure 1. Methods 1 and 2 seem to have produced composites with some agglomeration of the nanofibers, but with a relative good distribution of the clusters (Figure 1, top left and top right). Method 3 yields a homogeneous mix (Figure 1, bottom left). Conversely, Method 4 generates poor dispersion and the worst distribution as compared with the other methods (Figure 1, bottom right). The large clusters are hollow, with the matrix clearly visible in their interior. The concept of dispersion is related to the formation of filler agglomerates/clusters in the domain; a good dispersion implies the fillers are well separated in the domain. In this study, we also consider the distribution of agglomerates/clusters in the domain; a uniform distribution of the agglomerates/clusters throughout the matrix is said to be a good cluster distribution. A sketch of distribution and dispersion concepts can be found in [3].

**Figure 2:**
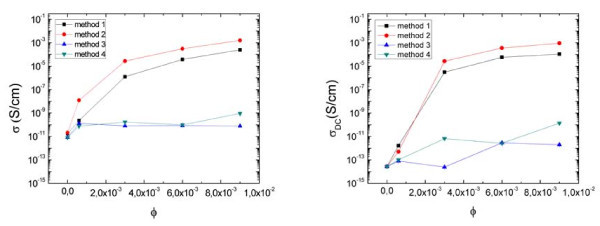
**Left-AC conductivity (σ) at 1 kHz versus volume fraction (*ϕ*) displayed in a log-linear scale**. Right-DC conductivity (σ_DC_) versus volume fraction (*ϕ*) displayed in a log-linear scale.

Figure 2 shows the AC conductivity at 1 kHz (left) and the DC conductivity (right) for different volume fractions. Depending on the method of composite preparation, a distinct conductivity behavior is observed. Samples prepared by Methods 1 and 2 reveal a dramatic increase in the DC conductivity of 6 and 8 orders of magnitude (Figure 2, right), respectively, between 0.0006 and 0.003 volume fraction. Methods 3 and 4 generate samples with low conductivity that is almost independent of the volume fraction. The jump of conductivity between 0.0006 and 0.003 volume fraction is also observed for the AC measurements (Figure 2, left). These results indicate that the percolation threshold can be found between 0.0006 and 0.003 volume fraction for the composites obtained with Methods 1 and 2, and at higher volume fractions for those obtained with Methods 3 and 4.

For fibers with a capped cylinder shape, the theoretical framework developed by Celzard [15], based on the Balberg model [16], provides the bounds for the percolation threshold. In general, the percolation threshold is defined within the following bounds:(1)

Equation 1 links the average excluded volume, 〈*V*_e_〉, i.e., the volume around an object in which the center of another similarly shaped object is not allowed to penetrate, averaged over the orientation distribution, with the critical concentration (*Φ*_c_), where 1.4 corresponds to the lower limit-infinitely thin cylinders-and 2.8 corresponds to spheres. These values were obtained by simulation. Using the values provided by the manufacturer of the VGCNF used in this study [4], Equation 1 predicts the bound 2E-3 ≤ *Φ*_c _≤ 3E-3 for an average aspect ratio of 433. The *Φ*_c _found in this study for Methods 1 and 2 (6E-4 <*Φ*_c _< 3E-3) includes the predictions of the theory, with exception of the upper bound. This indicates that a network is formed, but it does not necessarily imply a physical contact between the VGCNF, as demonstrated in [9,12].

**Figure 3:**
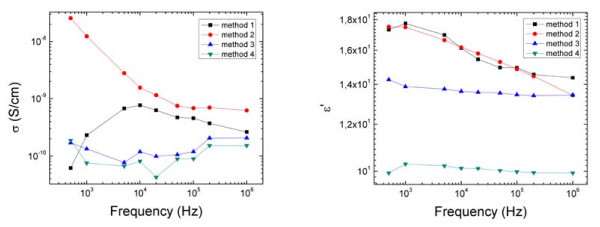
**Left-conductivity versus frequency for the four methods**. Right-dielectric constant versus frequency for the four tested methods. Results are for the 0.006 volume fraction sample.

Figure 3 (left) shows the measured AC conductivity of the four composites for a range of frequencies. The conductivity of composites prepared by Methods 1 and 2 is more strongly dependent on frequency. Figure 3 (right) presents the dielectric constant versus frequency for the methods under investigation, for a volume fraction of 0.006. Again, the dielectric constant shows a larger frequency dependency for composites 1 and 2.

By relating the electrical response (Figures 2, 3) with the level of mixing of the VGCNF in the matrix (Figure 1), it appears that the samples with better VGCNF dispersion exhibit the lowest conductivity. A better cluster distribution results in lower percolation threshold and higher conductivity for a given volume fraction.

## Conclusions

Four dispersion methods were used for the preparation of VGCNF/epoxy composites. It is shown that each method induces a certain level of VGCNF dispersion and distribution in the matrix, and that these have a strong influence on the composite electrical properties. A homogenous VGCNF dispersion does not necessarily imply better electrical properties. In fact, it seems that the presence of well-distributed clusters is more important for the electrical properties, which is in agreement with the experimental results of [13] for MWCNT/polymer composites.

These results provide important insights into the usefulness of each method. More importantly, they improve our understanding of the relationships between VGCNF dispersion and the electrical properties, which is an important step to pave the way for further research into tailoring the properties of these nanocomposites for specific applications.

## Abbreviations

CNT: carbon nanotubes; CAS: chemical abstract service; SEM: scanning electron microscopy; VGCNF: vapor-grown carbon nanofibers.

## Competing interests

The authors declare that they have no competing interests.

## Authors' contributions

PC carried out the conductivity studies, participated in the SEM analyses and participated in the writing of the manuscript. JS participated in the SEM analysis, theoretical interpretation and drafted the manuscript. JC conceived and designed the Method II of this study and participate in writing the manuscript. DK conceived and designed methods III and IV and participated in writing the manuscript. FWJH, RJS and SLM designed and coordinated the study, lead the discussion of the results and participated in writing the manuscript. All authors read and approved the final manuscript.
